# The principle of magnetic flux switch

**DOI:** 10.1038/s41598-024-59721-0

**Published:** 2024-04-18

**Authors:** Amir Heidary, Mohammad Ghaffarian Niasar, Marjan Popov

**Affiliations:** Faculty of EEMCS, Delft University of Technology, Mekelweg 4, 2628CD Delft, The Netherlands

**Keywords:** Engineering, Electrical and electronic engineering

## Abstract

This paper introduces an innovative Magnetic Switch (MFS) designed to control and alter magnetic flux within energy system components, offering an alternative to conventional power electronic devices. The MFS comprises a low-current control coil, a control core, and a high-density magnetic flux-carrying main core combined with a main coil energy system. In this novel magnetic configuration, when a low-power current excites the control coil, the magnetic flux in the main core (supplied by the main coil) decreases to nearly zero. Conversely, when the control coil disconnects from the power source, the magnetic flux within the main core attains its maximum value. This operation positions the MFS as a groundbreaking concept within magnetic-based energy systems, akin to transistors in power electronics. The main outcomes of the MFS concept are that it can vary the magnetic flux of the main core in the large range, and it is a fast magnetic switch with a simple and low power loss control circuit and an independent control coil from the main coil. Analytical studies thoroughly elucidate the performance and advantages of this proposed magnetic switch, substantiated by Finite Element Method simulations and experimental prototype outcomes.

## Introduction

Previously, The control of the magnetic flux in a ferromagnetic core via an external controller has been investigated by several applications^1^. This application proposes to change the value of the main magnetic flux in the ferromagnetic arm to control the behavior of electric machines like reactors and transformers. A magnetic amplifier (MA) saturable-core controllable reactor as a concept of magnetic flux controller was developed for the first time in 1920^1^. The functionality of the MA is analogous to the electric field in the field-effect transistors (FET)^2^. An MA consists of a magnetic core with a main and controlling DC coil. The main current of the circuit passes through the main coil, which is wounded on the core leg. Consequently, the main coil magnetomotive force generates the core's main flux. The DC controlling coil is wound on the core's other leg and driven by a DC low current/voltage^2^. Therefore, the amount of the main magnetic flux is controlled by DC controlling magnetic flux. So far, many different structures have been developed based on the MA concept, such as controllable transformers^3^, controllable reactors^4^, fault current limiters^5^, and power flow control mechanisms^6^. The following issues are observable in MA:

- The MA control system consumes energy because of its direct face with the main flux of the core.
- DC control flux in the core is in the common path with the main flux, affecting the main flux's magnitude.
- Induced voltage in the DC control coil by the main flux variation affects the performance of the controlling process.

On the other hand, a magnetic flux valve (MFV) was introduced in ^7^. It is mainly used in magnetic circuits to control magnetic flux. The MFV contains a laminated structure of two different types of layers made of magnetostrictive material, e.g., amorphous alloy ribbon, and piezoelectric material, e.g., piezoelectric sheet. The permeability of the magnetostrictive layers changes when the external control voltage, which is applied to the piezoelectric layer, changes. This phenomenon is known as the converse magnetoelectric effect^7^.

The main difference between MA and MFV is how the main magnetic flux of the core is controlled^2,7^. MFV is used to build an adjustable ratio transformer^8,9^. Moreover, in ^10^, as with other applications of MFV, a tunable reactor is presented for electric systems. The following issues are observable in MFV:

- MFV has a complex structure consisting of the electrode, piezoelectric, and magnetostrictive layers.
- The magnetic permeability of electrodes and piezoelectric layers is very low, and these two layers are replaced instead of ferromagnetic material in the path of the core magnetic flux.
- Minimum reluctance of MFV is higher than that in the main core with the same length.
- MFV affects the magnetic characteristics of the main core and decreases its performance.
- The thermal coefficients of ferromagnetic materials, piezoelectric materials, and electrodes are different and cause mechanical stress via temperature changes.
- MFV will be saturated faster than the main core because of the lower cross-section of the ferromagnetic materials.

Furthermore, orthogonal fluxgate (OFG)^11^ is a concept in which operations are based on saturating magnetic flux and are mostly used as a sensor to measure the magnetic flux. In the structure of the OFG^12^, the flux direction of the output coil consists of the piece of core and a large air direction. Therefore, the inductance of this coil is very low in both the saturation and non-saturation regions of the core, and it has a very minor effect on the flux of the core^13^. Consequently, it can not behave like a transistor, which has significant effects on core magnetic flux.

This paper proposes a novel structure that can switch the magnetic flux of the main ferromagnetic arm, which is called the magnetic flux switch (MFS). This component can switch the magnetic flux from near zero to maximum, considerably improving the control of magnetic-based machines^14^. In the proposed structure, the switching action of the main core flux is performed by controlling the DC flux of a control core, which flows in a separate core. Indeed, control will be possible by the small excitation current of the control coil because the main and control fluxes do not flow in a common path. The power loss of the proposed MFS is independent of the value of the main flux. The main advantages of the proposed Magnetic flux switch over the state-of-the-art are as follows:

- High magnetic flux variation ratio
- Low trigging voltage and current
- Low trigging power loss
- Simple control system
- High switching speed
- No mutual inductance between the main and control coil

## Results and discussion

### The design principle of MFS

**Figure 1:**
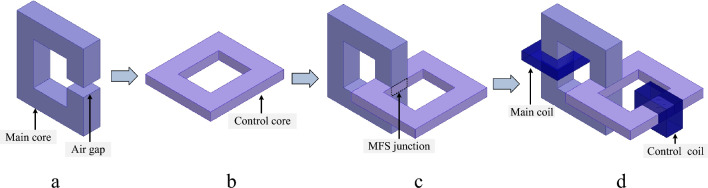
MFS assembly and configuration. (**a**) Main core. (**b**) Control core. (**c**) Cores combination. (**d**) MFS complete structure.

In this section, the principles and configuration of the proposed MFS are presented. As shown in Fig. 1, the proposed structure consists of a main ferromagnetic, a main coil, a control ferromagnetic core, and a control coil. In Fig. 1a, the main core is shown. The control core is depicted in Fig. 1b. Figure 1c shows the assembly of the MFBS. The connection between vertical and horizontal cores forms the MFS junction. Lastly, in Fig. 1d, the complete structure of the MSF is presented in which the main coil is wounded on the main core and a DC excited coil as the control coil is wound on the control core.

### MFS operation

**Figure 2:**
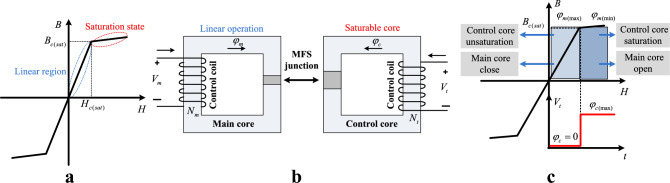
MFS operation regions. (**a**) B-H characteristic of cores. (**b**) MFS structure. (**c**) MFS operation regions.

In this structure, it is possible to switch the magnetic flux of the main core. The main core flux changes from a negligible value to its maximum. This main flux is generated by the main coil's magnetomotive force (mmf) and is passed through the main core and MFS junction. The switching process is carried out by applying an excitation pulse to the control coil. The MFS is at a closing state when the control coil is not energized, and the control core flux is zero. Therefore, all the generated flux by the main coil passes through the main core and the MFS junction. The MFS is at the opening state when the control coil is energized, and deep saturation of the control core occurs. Therefore, the flux of the main core cannot pass through the MFS junction, and its value reaches zero. Figure 2 illustrates the operation of MFS based on the magnetic characteristic of the control core and its B-H curve, where Fig. 2a is the magnetic characteristic of the control core, (b) is the disassembled view of the MFS, and (c) shows the operation of the MFS based on control pulse.

### Analytical study of MFS

**Figure 3:**
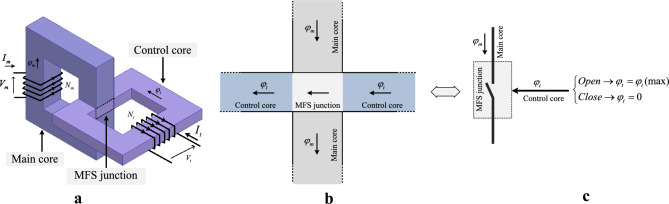
MFS structure and operation. (**a**) MFS assembled structure. (**b**) MFS junction and flux directions. (**c**) Junction operation as switch depends on control magnetic flux.

**Figure 4:**
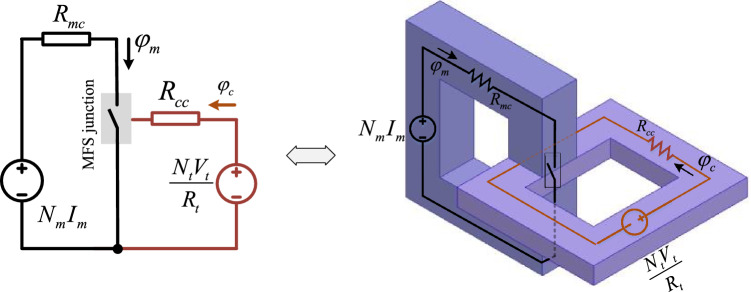
Electrical equivalent circuit of MFS structure.

This section uses the equivalent circuit of the proposed MFS to conduct analytical studies. The MFS assembled view of the magnetic structure is shown in Fig. 3a, where Fig. 3b magnifies the flux of the core and MFS junction. Figure 3c presents that the MFS junction can be modeled as a switch that operates in two states: open state when the control flux saturates the control core and closed state when the control core flux is zero. In Fig. 4, an electrical equivalent circuit of MFS is presented. Considering that the proposed MFS behaves analogously to an electronic switch, its equivalent magnetic circuit is more likely to convert to conventional electronic switches. In the proposed MFS, the magnetomotive force of the main coil is considered a voltage source, and ferromagnetic core reluctance is considered a series resistance with the voltage source. The MFS junction is used to switch the main core flux by the control flux of the control core, which is modeled as a switch.

The control core and control coil are the trigger circuits modeled as the MFS gate, which consists of a voltage source (magnetomotive force of control coil) and the reluctance of the control core. The MFS gate is excited by applying a voltage to the control coil. Considering the control coil's high inductance and high resistance, voltage and a very low current are needed to control the MFS. In the closing state, MFS imposes a very low reluctance considering the electromagnetic characteristic of the control core. MSF imposes a high reluctance in the MFS open state considering the deep saturation control core. Table 1 presents the modeling parameters data.

**Table 1:** Equivalent circuit parameters.

Parameter	Description
*N*_*m*_	The main coil turns
*N*_*t*_	The control coil turns
*I*_*m*_	Main coil current
*I*_*t*_	Control coil current
*V*_*t*_	Control coil voltage
*R*_*t*_	Control coil resistance
*R*_*mc*_	Main core reluctance
*R*_*cc*_	Control core reluctance linear part
*R*_*sat*_	Control core reluctance saturation state
*R*_*so*_	MFS reluctance (open state)
*R*_*sc*_	MFS reluctance (close state)
*ϕ*_*m*_	Main core magnetic flux
*ϕ*_*c*_	Control core magnetic flux
*µ*_*c*_	Control core magnetic permeability
*µ*_*sat*_	Control core saturation magnetic permeability
*µ*_*m*_	Main core magnetic permeability
*l*_*c*_	Control core average length
*l*_*m*_	Main core average length
*l*_*j*_	Length of cores junction
*L*_*t*_	Control coil linear inductance
*L*_*sat*_	Control coil saturation inductance

**Figure 5:**
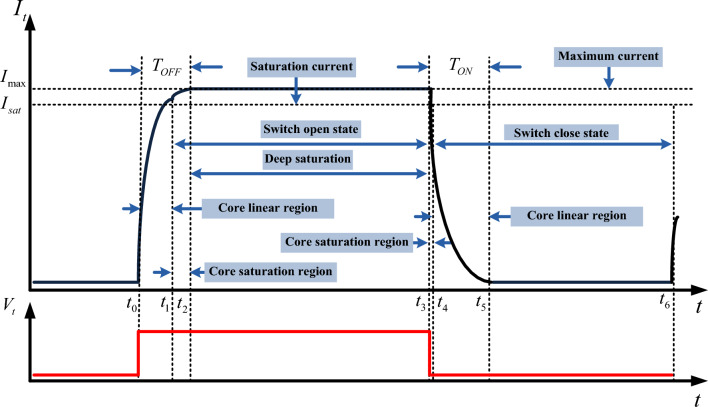
Trigger coil current rising and falling times (T_OFF_, T_ON_).

To show the operational delay of the switch as *T*_ON_ and *T*_OFF_, Fig. 5 presents the current rise and drop time constant. The control coil is excited by the trigger pulse. During *t*_0_–*t*_3_, the trigger pulse is at a high level, and during t_3_–t_6_ it is at a zero level. Raising *I*_*t*_ between *t*_0_–*t*_1_ shows the control coil current increase in the core linear region, and *t*_1_–*t*_2_ shows the current increase when the core is in the saturated region. During *t*_2_-*t*_3_ the current of the control coil reaches its maximum where the core is in the deep saturation region. By the interruption of the excitation source, in *t*_3_-*t*_4_, the current of the control coil drops when the core is still in the saturated region, and during *t*_4_-*t*_5_, the current of the control coil drops when the core is in the linear region. Consequently, the proposed MFS turning-off delay is calculated as *T*_OFF_ = *t*_0_ + *t*_2,_ and its turning-on delay is calculated as *T*_ON_ = −*t*_3_ + *t*_5_.

### MFS close state

In this state, the control coil is not energized, and consequently, the control coil current and control core magnetic flux are zero. In this condition, all of the main core magnetic flux is passed by the main core and magnetic junction. The main core Magnetic flux equation is:

1 $$\varphi_{mc} = k_{1} I_{m} \quad t_{4} > t > t_{6}$$

where: $$k_{1} = \frac{{N_{m} \mu_{m} A_{m} }}{{l_{m} }}$$.

This analysis considers that both the reluctance of the main core and MFS junction are linear. According to Eq. (1), the maximum magnetic flux generated by the main coil passes through the MFS junction. Considering the low length of the MFS junction, the value of magnetic path reluctance is approximately equal to the main core reluctance. Therefore, the main coil flux value depends on the current of the main coil and the main core reluctance.

### MFS open state

In this state, the control coil is energized, and the trigging voltage is equal to its maximum value then the control core is deeply saturated by the control coil current. Considering core junction deep saturation, the main core magnetic flux significantly decreases, and its value reaches zero. Equation (2) shows the magnetic flux of the main core as follows:

2 $$\varphi_{mo} = \frac{{N_{m} I_{m} }}{{R_{mc} + R_{so} }} = \frac{{N_{m} I_{m} }}{{\frac{{l_{j} }}{{A_{m} \mu_{c - sat} }}}} = \left( {\frac{{A_{m} N_{m} }}{{l_{j} }}} \right) I_{m} \mu_{sat} \quad t_{1} > t > t_{3}$$

where: $$R_{mc} < < R_{so}$$.

Therefore, by decreasing the main core flux close to zero, the MFS high reluctance behaves as an open switch.

### MFS operation ratio

The Computation of Eq. (3) has been included to illustrate the flux variation ratio in the MFS. This equation, designated by the division of Eqs. (1) to (2), calculates the rate of main coil flux variation from open to closed states.

3 $$MFR = \frac{{\varphi_{mc} }}{{\varphi_{mo} }} = \frac{{\frac{{N_{m} \mu_{m} A_{m} }}{{l_{m} }} \cdot I_{m} }}{{\left( {\frac{{A_{m} N_{m} }}{{l_{j} }}} \right) I_{m} \mu_{sat} }} = \frac{{l_{j} \mu_{m} }}{{l_{m} \mu_{sat} }}$$

Upon observing the equation above, *MFR*, denoted as the magnetic flux ratio variation of the MFS, elucidates the extent of magnetic flux change from its maximum to minimum values, which amounts to a considerable range. Moreover, this range can be further augmented by extending the length of the MFS junction and enhancing the permeability of the core magnetic flux.

### Magnetic flux of the control core

The change of *φ*_*m*_ is expressed considering Eq. (4) by exciting the control coil by the direct current. These equations describe the value of the control core flux in the linear, saturation, and deep regions.

4 $$\varphi_{c} = \left\{ \begin{aligned} & K_{3} I_{t} \to Linear{\text{-}}Region \hfill \\ & K_{4} I_{sat} \to Saturation{\text{-}}Region \hfill \\ & K_{5} I_{\max } \to Deep{\text{-}}Saturation{\text{-}}Region \hfill \\ \end{aligned} \right.$$

where: $$K_{3} = \frac{{A_{c} }}{{l_{c} }}\mu_{c} N$$ $$K_{4} = \frac{{A_{c} }}{{l_{c} }}\mu_{sat} N_{t}$$ $$K_{5} = \frac{{A_{c} }}{{l_{c} }}\mu_{Dsat} N_{t}$$.

The first term of the magnetic flux in (4) depends on the control core reluctance in the linear region, and the second term calculates magnetic flux in the saturation region. The last term of the control core magnetic flux is obtained considering core reluctance in the deep saturation region.

### Control coil current dynamics

**Figure 6:**
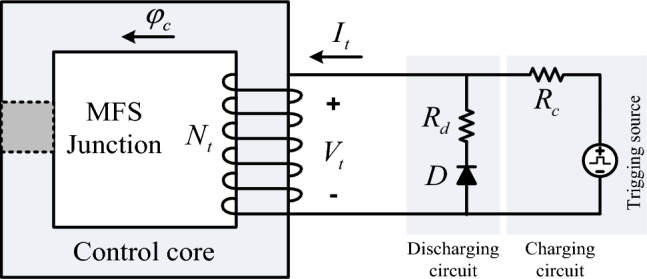
Trigger section electric circuit.

The analysis of the trigging current dynamics determines the possible switching frequency of MFS. Figure 6 illustrates the dynamic of the control coil current. This figure shows that Rc connects the control core to the trigging source. The diode and Rd discharge the current of the control coil (*I*_*t*_). The dynamic equation is written in current charging and discharging states as Eqs. (5) and (6).

5 $$i_{t} (t) = \left\{\begin{array}{lll} \frac{{V_{tr} }}{{R_{t} + R_{c} }}(1 - e^{{ - \alpha_{1} t}} ) & \quad Linear{\text{-}}Region & \quad t_{0} < t < t_{1} \hfill \\ \frac{{V_{tr} }}{{R_{t} + R_{c} }}(1 - e^{{ - \alpha_{1} t_{1} }} ) + \frac{{V_{t} }}{{R_{t} + R_{c} }}(1 - e^{{ - \alpha_{2} (t - t_{1} )}} ) & \quad Saturation{\text{-}}Region & \quad t_{1} < t < t_{2} \hfill \\ \frac{{V_{tr} }}{{R_{t} + R_{c} }} & \quad Deep{\text{-}}Saturation{\text{-}}Region & \quad t_{2} < t < t_{3} \hfill \\ \end{array} \right.$$

6 $$i_{t} (t) = \left\{ \begin{array}{lll} \frac{{V_{tr} }}{{R_{t} + R_{c} }}(e^{{ - \alpha_{3} (t - t_{3} )}} ) & \quad Saturation{\text{-}}Region & \quad t_{3} < t < t_{4} \hfill \\ \frac{{V_{tr} }}{{R_{t} + R_{c} }}(e^{{ - \alpha_{3} (t_{4} - t_{3} )}} )(e^{{ - \alpha_{4} (t - t_{4} )}} ) & \quad Linear{\text{-}}Region & \quad t_{4} < t < t_{5} \hfill \\ \hfill \\ 0 & \quad Swith{\text{-}}closed{\text{-}}State & \quad t_{5} < t < t_{6} \hfill \\ \end{array} \right.$$

where: $$\alpha_{1} = \frac{{l_{c} }}{{A_{c} \mu_{c} }}\frac{{R_{t} + R_{c} }}{{N_{t}^{2} }}$$ $$\alpha_{2} = \frac{{l_{c} }}{{A_{c} \mu_{c - sat} }}\frac{{R_{t} + R_{c} }}{{N_{t}^{2} }}$$ $$\alpha_{3} = \frac{{l_{c} }}{{A_{c} \mu_{c - sat} }}\frac{{R_{t} + R_{d} }}{{N_{t}^{2} }}$$ $$\alpha_{4} = \frac{{l_{c} }}{{A_{c} \mu_{c} }}\frac{{R_{t} + R_{d} }}{{N_{t}^{2} }}$$

7 $$T_{OFF} = t_{2} = \left( { - \frac{1}{{\alpha_{1} }}\ln ( - \frac{{i_{t} (t_{1} )(R_{t} + R_{c} )}}{{V_{tr} }} + 1) - \frac{1}{{\alpha_{2} }}\ln ( - \frac{{i_{t} (t_{2} ) - i_{t} (t_{1} )(R_{t} + R_{c} )}}{{V_{tr} }} + 1)} \right)$$

8 $$T_{ON} = - t_{3} + t_{5} = \left( { - \frac{1}{{\alpha_{3} }}\ln (\frac{{i_{t} (t_{4} )(R_{t} + R_{c} )}}{{V_{tr} }}) - \frac{1}{{\alpha_{4} }}\ln (\frac{{i_{t} (t_{5} )}}{{i_{t} (t_{4} )}})} \right)$$

Here, Rt is the resistance of the control coil, and *R*_*c*_ is the external control source series resistor. Considering time constant ${\alpha }_{1}$ and ${\alpha }_{2}$ The control coil's charging and discharging delay defines the *T*_*OFF*_ and *T*_*ON*_ value as MFS operational delay. Equations (7) and (8) show that MFS turning-off and on delay are calculated considering the control coil charging and discharging current.

To compute the maximum power loss in the control circuit *P*_*loss-c*_ the Eq. (9) can be driven:

9 $$P_{loss{\text{-}}c} = \frac{{V_{tr}^{2} }}{{\left( {R_{t} + R_{c} } \right)}}$$

This equation illustrates that the power loss of the MFS control circuit relies on the resistance of the control coil, the internal and external resistance of the triggering circuit, and the triggering voltage. Given that the magnitude of the triggering voltage is low and the resistance of the circuit is high, the power loss value will be minimal.

### Mutual inductance of the main and control coil

The crucial feature of the MFS that needs particular attention is the mutual inductance between the main and control coils. The Eq. (10) governing the mutual inductance between the coils is as follows:

10 $$M = k\sqrt {L_{m} \cdot L_{c} }$$

M is mutual inductance, k is coupling coefficient, and *L*_*m*_ and *L*_*c*_ are main and control coil inductances, respectively. By extension of this equation, the following Eq. (11) is obtained:

11 $$M = \frac{{\varphi_{c} }}{{\varphi_{m} }}|_{{i_{c} = 0}} \sqrt {\left( {\frac{{\varphi_{m} N_{m} }}{{i_{m} }}} \right)\left( {\frac{{\varphi_{c} N_{c} }}{{i_{c} }}} \right)}$$

In the equation above, it is noted that when the control current approaches zero, the rate of control core flux to main core flux also diminishes significantly. Consequently, the mutual inductance between the control and main coils tends toward zero. This characteristic enables the MFS to function as an isolated gate magnetic switch, presenting a unique advantage. We have included these equations and corresponding explanations in the manuscript.

### MFS operation in the AC mode

**Figure 7:**
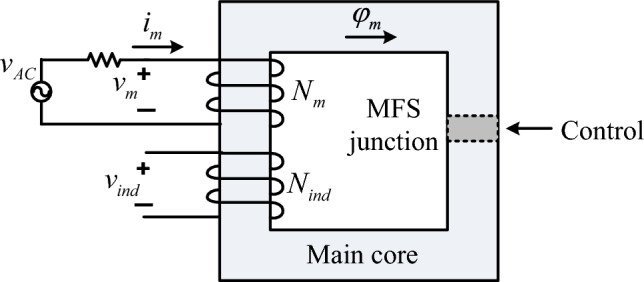
AC operation of MFS.

In this section, it is considered that the main coil is connected to the sinusoidal source, and induced voltage on the secondary coil to the main core is analyzed (Fig. 7). As depicted in this figure, and the main coil is connected to the AC source by resistance and the induced voltage of the secondary coil will be analyzed. On the other hand, the main core flux is controlled by MFS junction reluctance.

The flux of the main core is (12), and the main coil current equation is presented in (13). In this analysis, the operation of MFS is studied where a secondary coil is wounded on the main core. Therefore, due to MFS switching, secondary coil voltage induction appeared. By substituting (13) in (12), the main core flux becomes (14). Finally, the induced voltage on the secondary coil *v*_*ind*_ is calculated as (15), where *N*_*ind*_ is the number of the secondary coil turns.

12 $$\varphi_{m} = \frac{{N_{m} i_{m} }}{{R_{mc} + R_{MFS} }}$$

13 $$i_{m} (t) = I_{\max } \sin (\omega t)$$

14 $$\varphi_{m} (t) = \frac{{N_{m} I_{\max } \sin (\omega t)}}{{R_{m} + R_{MFS} }}$$

15 $$\begin{aligned} v_{ind} (t) & = - N_{ind} \frac{d}{dt}\varphi_{m} = - N_{ind} \frac{d}{dt}\left( {\frac{{N_{m} I_{\max } \sin (\omega t)}}{{R_{m} + R_{MFS} }}} \right) \\ & = \frac{{\omega N_{ind} N_{m} I_{\max } \cos (\omega t)}}{{R_{m} + R_{MFS} }} \hfill \\ \end{aligned}$$

### FEM simulation of MFS

This section presents the obtained results by using electromagnetic FEM analysis to illustrate the electromagnetic behavior of the proposed structure. Designed MFS parameter data are presented in Table 2.

**Table 2:** Parameters of FEM simulation.

Parameter	Description	Value
*L*_*m(close)*_	Main coil linear inductance (MFS close state)	500 mH
*L*_*m(open)*_	Main coil linear inductance (MFS open state)	10 mH
*L*_*t*_	control coil linear inductance	500 H
*L*_*sat*_	control coil saturation inductance	3 mH
*I*_*m*_	Main coil current	50 mA
*I*_*t*_	Control coil current	18 × 10^–6^ A
*V*_*t*_	Control coil voltage	15 V
*R*_*t*_	Control coil resistance	0.83 MΩ
*µ*_*c*_	Control core magnetic permeability	4000 × 4π × 10^–7^
*µ*_*sat*_	Control core saturation magnetic permeability	1.3 × 4π × 10^–7^
*µ*_*m*_	Main core magnetic permeability	4000 × 4π × 10^–7^
*l*_*c*_	Control core average length	12 cm
*l*_*m*_	Main core average length	12 cm
*l*_*j*_	Length of cores junction	0.5 cm

**Figure 8:**
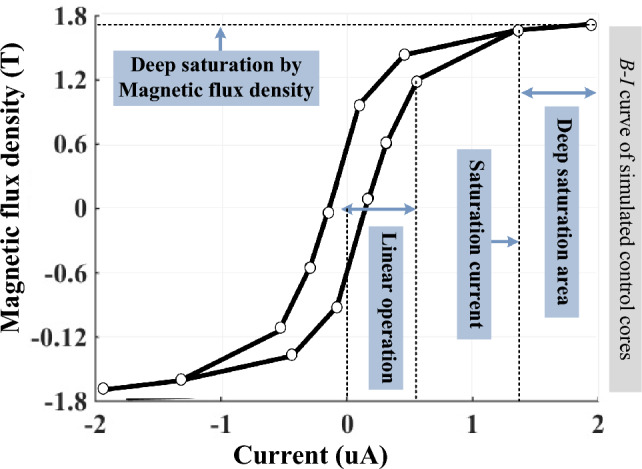
Magnetic characteristic of the control core.

Figure 8 shows the magnetic characteristic of the control core. The core deep-saturation occurs by feeding trigger current higher than 1.4 × 10^–6^ A.

**Figure 9:**
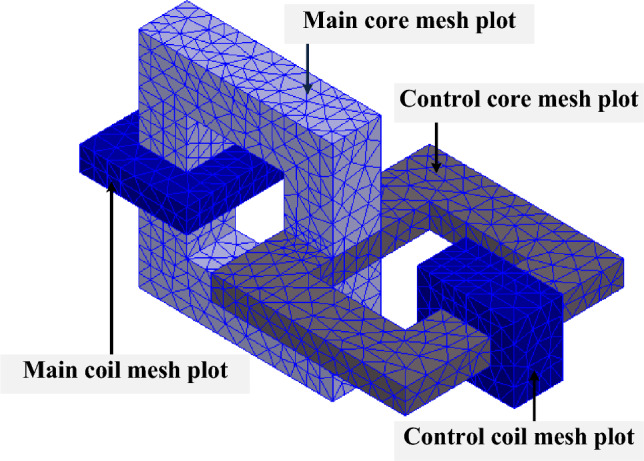
Mesh plot of the simulated MFS.

In this section, transient analysis of FEM simulation is reported. The control coil is excited by a pulse voltage with a 50% duty cycle and 1 kHz. Moreover, the main coil is connected to the DC source and consists of internal resistance. Figure 9 depicts the mesh plot of the three-dimensional design of MFS.

### Simulation of MFS in the open state

**Figure 10:**
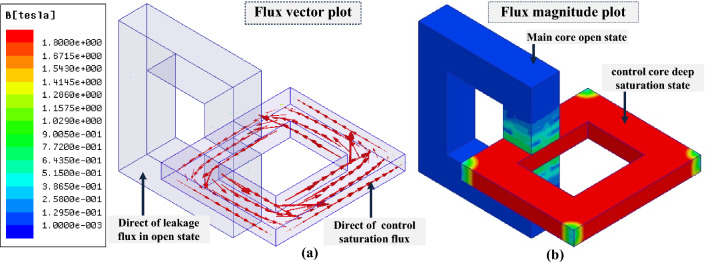
FEM simulation of MFS (switch off-state). (**a**) Direction of magnetic flux. (**b**) Magnetic flux plot on the section surface.

In this section, MFS off-state operation is simulated. It is evident that the saturation of the control core results in the main core flux declining nearly to zero. Therefore, by applying proper energization commended to the control coil, the main core's magnetic flux will decrease close to zero, and MFS will behave as an open switch. In Fig. 10, the FEM simulation of MFS is presented in the switch off-state by depicting the direction of magnetic flux in Fig. 10a and its average value plot on the section surface in Fig. 10b. The magnetic flux of the control core is 1.8 T, and the magnetic flux of the main core reaches nearly zero while the main coil is excited by the DC source. Moreover, in this state, the inductance of the main coil is mostly related to the leakage flux of the coil, which, in this model, reaches the maximum value of 10 mH and is independent of the magnetic flux of the main core.

### Simulation of MFS in the close state

**Figure 11:**
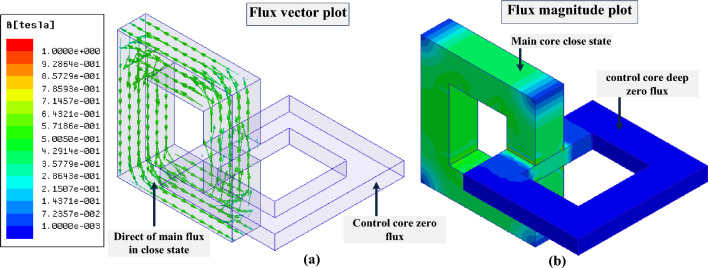
FEM simulation of MFS (switch on-state). (**a**) Direction of magnetic flux. (**b**) Magnetic flux plot on the section surface.

In the close state, the control coil is not excited. Consequently, the magnetic flux of the control core coil is zero. Therefore, MFS is in the on-state, and the magnetic flux of the main core exceeds its maximum value. Figure 11 shows the FEM simulation result while MFS is in the on-state. In this figure, it is depicted that the magnetic flux of the control core reaches zero, and all the magnetic flux passes through the main core and cores junction (MFS junction). The main coil flux exceeds almost 0.7 T in the MFS on-state; the mmf of the main coil generates this magnetic flux.

The simulation results show that the ratio consisting of the division of the main core flux in the closed state to the open state (700 mT/0.7 mT) equals 1000. This ratio proves the huge variation of flux in the main core between two different states and the acceptable performance of the MFS.

### DC operation of MFS

**Figure 12:**
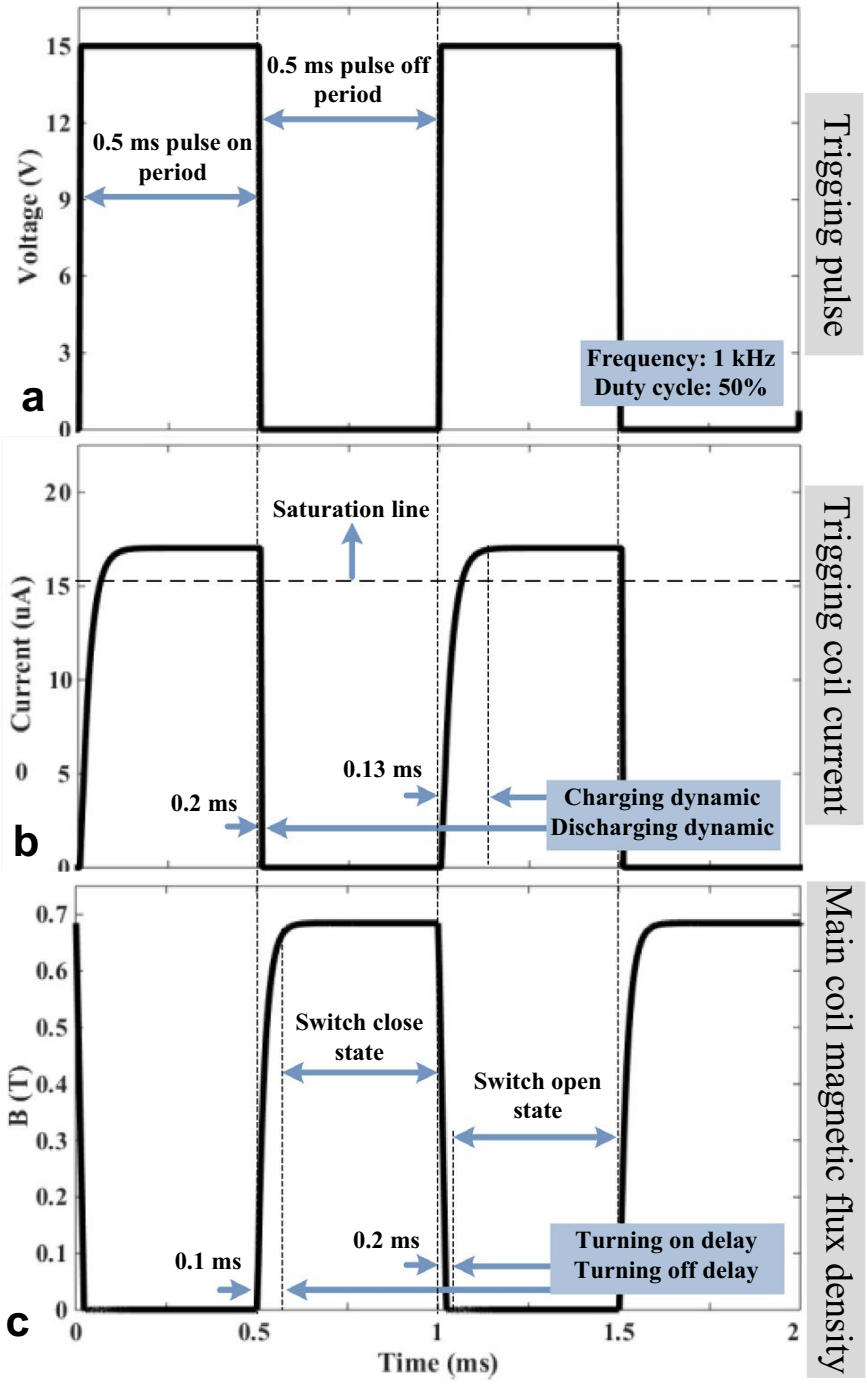
FMS DC FEM simulation. (**a**) Trigging source voltage. (**b**) Control coil current. (**c**) Main coil magnetic flux density.

In transient FEM analysis, magnetic flux, currents, and voltages are obtained considering MFS switching operation using a 1 kHz trigging voltage source. In this analysis, signal dynamics and continuous operation of MFS are presented. Figure 12 shows the MFS signals in the switching operation.

Figure 12a shows the trigging pulse that generates a control pulse. The magnitude of this pulse is 15 V, and its frequency and duty cycle are 1 kHz and 50%. The current of the control coil is shown in Fig. 12b, where the maximum value reaches 17 *µ*A. As a result, the magnetic flux of the main coil is switched, and its signal is depicted in Fig. 12c. In the main core, the maximum magnetic flux density exceeds 0.7 T, and its minimum reaches nearly zero, showing the proper operation of the MFS.

### Bidirectional AC operation of MFS

**Figure 13:**
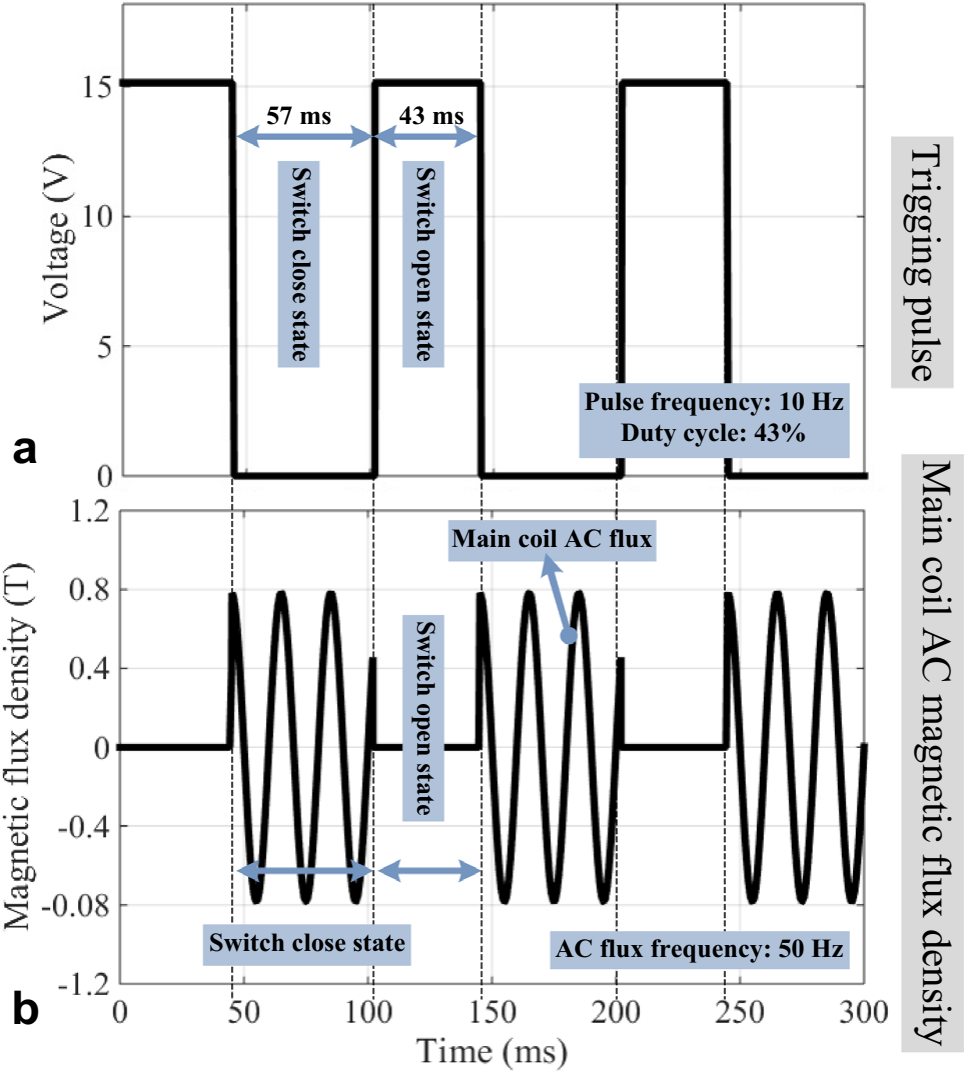
FMS AC FEM simulation. (**a**) Trigging source voltage. (**b**) Main core AC flux.

Connecting the AC source to the main coil makes it possible to illustrate the operation of the MFS while AC magnetic flux passes through the main core. In this case, the AC flux of the main core is switched by applying the control pulse to the control coil, as shown in Fig. 13a. The controlling pulse duty cycle is 43%, and its frequency is 10 Hz. By applying 15 V to the control coil, MFS changes into an open state, and the AC flux of the main core declines to zero.

By applying zero voltage to the control coil, MFS closes the AC flux path, and the main core AC flux value exceeds its maximum. As shown in Fig. 13b, the peak value of the main core AC flux reaches 0.8 T. As demonstrated in this simulation, it is evident that MFS controls the AC flux of the main core as a bidirectional switch.

### Analogy of MFS with mature saturating devices

The specifications of MFS are comparable with some conventional components(Supplementary Information). These devices can control the value of the magnetic flux in the core based on core saturating like MA and OFG or piezoelectric like MFV. To show the performance of the MFS, the main specifications of MFS, such as inductance of the main coil in the closed state, Flux ratio (*φ*_m (closed)_/ *φ*_m (open)_), Switching operation frequency, The control circuit of the switch, Trigging voltage, Trigging voltage. The comparison results are presented in Table 3.

**Table 3:** Comparison of MFS with well-known saturating structures.

Specification	MFS	MA	MFV	OFG
The inductance of the main coil in the closed state	More than 500 mH	More than 500 mH	Less than 500 mH	Very small
Flux ratio ϕ_m(closed)/_ϕ_m(open)_	More than 1000	More than 1000	Very small	Very small
Switching operation frequency	1 Hz < f < 10 kHz	Very low	1 Hz < f < 1 MHz	1 Hz < f < 100 Hz
The control circuit of the switch	Control coil	Control coil	Piezoelectric	Control coil
Trigging type	Magnetic field effect	Magnetic field effect	Electric field effect	Magnetic field effect
Trigging voltage	*V*_*t*_ = 15–20 V	*V*_*t*_ = 100–1000 V	*V*_*t*_ = 15–30 V	*V*_*t*_ = 10–1000 V
Trigging current	*I*_*t*_ = 10–100 µA	*I*_*t*_ = 10–1000 A	*I*_*t*_ = 10–1000 mA	*I*_*t*_ = 10–1000 A

### MFS and IGBT analogy

**Figure 14:**
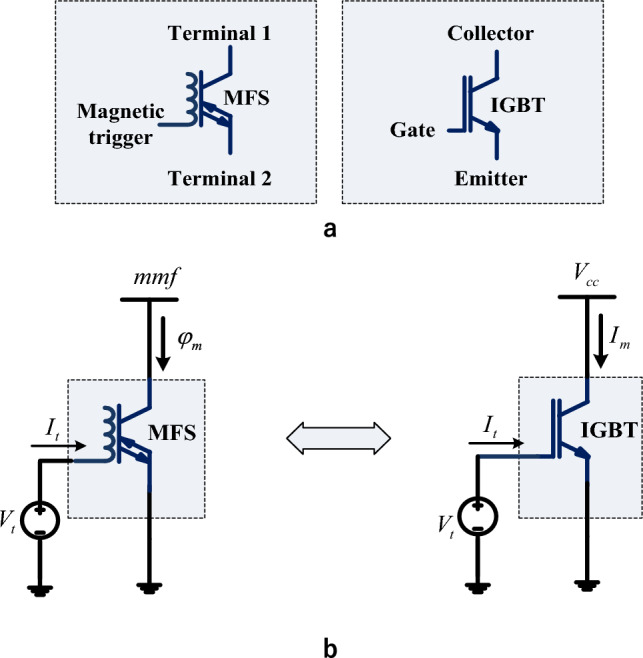
Comparison of MFS and IGBT. (**a**) MFS, and IGBT symbols. (**b**) Analogy of MFS and IGBT operation.

In this analogous study, an insulated gate bipolar transistor (IGBT) candidate from the field-effect transistor is vastly utilized as a power electronic switch in energy systems, for instance, in ^12^. The analogy of MFS and IGBT exposes the similarity of MFS and IGBT as a common type of industrial electronic switch; while their technology is far from each other, their main purpose is to control and alter magnetic flux in energy system ferromagnetic cores. Figure 14a shows symbols of the proposed MFS and IGBT. In Fig. 14b, the analogy of MFS and IGBT is illustrated. To explain the IGBT switch, in the switching operation mode, the collector terminal of the IGBT connects to a voltage source as *V*_*cc*_. By IGBT turning on, the current of source *I*_*m*_ passes through the collector to the emitter terminal. By IGBT turning off, *I*_*m*_ declines to zero. The gate terminal controls the IGBT switch, which is driven by applying control voltage *V*_*t*_. The gate of IGBT is modeled as a capacitive circuit, known as an electric field-effect transistor. Gate input current *I*_*t*_ is very low. The first MFS terminal is connected to mmf, modeled as a voltage source for expressing the MFS's switching operation. In the turning on the state, flux *φ*_m_ passes by MFS from terminal 1 to terminal 2. In the turning-off state, MFS interrupts *φ*_m,_ and its value declines to zero. The magnetic trigging terminal controls the MFS, which is connected to trigging voltage *V*_*t*_. This switch has a magnetic field effect, and the trigging curren_*t*_ is very low. Accordingly, a comparison of MFS and IGBT is presented in Table 4.

**Table 4:** Comparison of MFS and IGBT.

Specification	MFS	IGBT
Used construction technology	Ferromagnetic materials	Semiconductors
Supplied source	Magnetomotive force (*mmf* = *N*_*m*_*.I*_*m*_)	Voltage source (*V*_*cc*_)
Switched parameter	Magnetic flux ϕ_m_	Current *I*_*m*_
The control circuit of the switch	Inductive	Capacitive
Trigging type	Magnetic field effect	Electric field effect
Controlling source	Voltage source *V*_*t*_ = 15–20 V	Voltage source *V*_*t*_ = 15–20 V
Trigging current	Control coil current *I*_*t*_ = 10–100 µA	Gate current *I*_*t*_ = 0.1–1000 mA
Operational direction	Bidirectional	Unidirectional

### MFS experimental results

**Figure 15:**
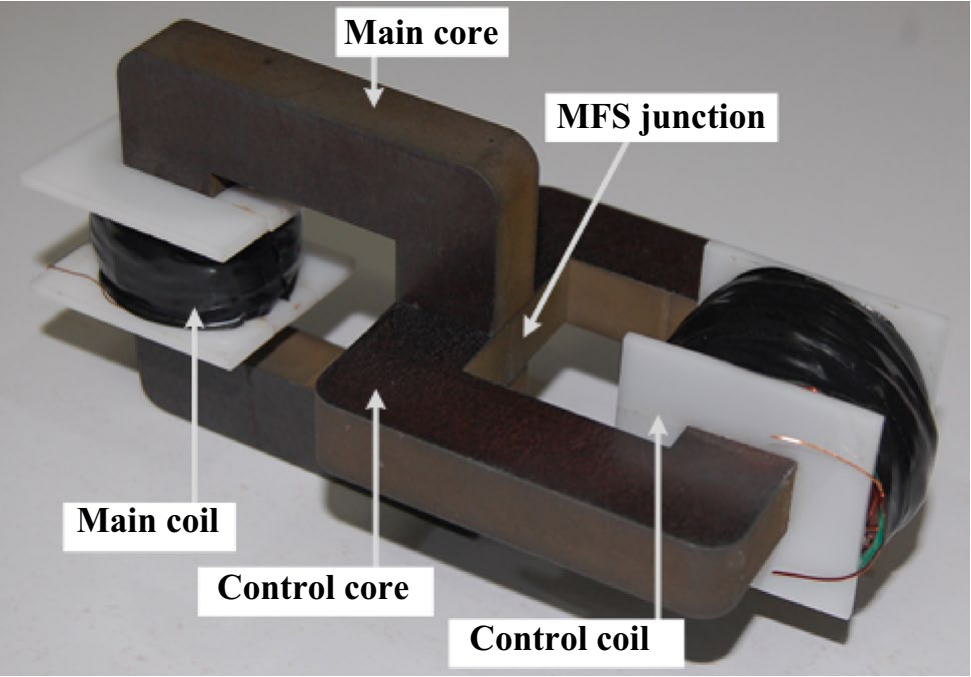
MFS laboratory prototype.

Figure 15 depicts the developed laboratory prototype of MFS. This prototype is implemented to validate the operation of MFS as a device that can switch magnetic flux.

**Figure 16:**
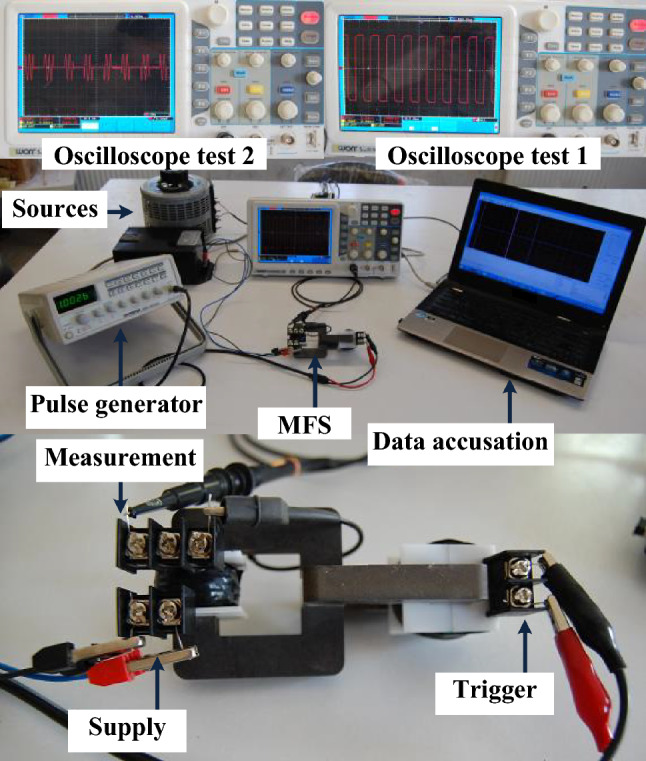
MFS laboratory test setup.

The design parameters of this prototype are presented in Table 2, which is similar to the simulation data. The designing procedure of the MFS prototype is as follows: first of all, ferromagnetic cores are created considering the geometric scheme and assembled together. Then, the control coil is wounded on the control core. Next, the main controlled coil is wounded on the main core to generate the main magnetomotive force. This prototype is used to confirm the operation of MFS in three different scenarios. The first scenario examines MFS's ability to switch the main core DC magnetic flux using a control pulse. The second scenario demonstrates MFS operation to control AC flux as a bidirectional switch. In the last scenario, a comparison is carried out between a boost DC/DC converter that is implemented on the one hand with an IGBT switch and, on the other hand, by MFS. Figure 16 illustrates the laboratory test setup.

### MFS DC magnetic flux switching test (first scenario)

**Figure 17:**
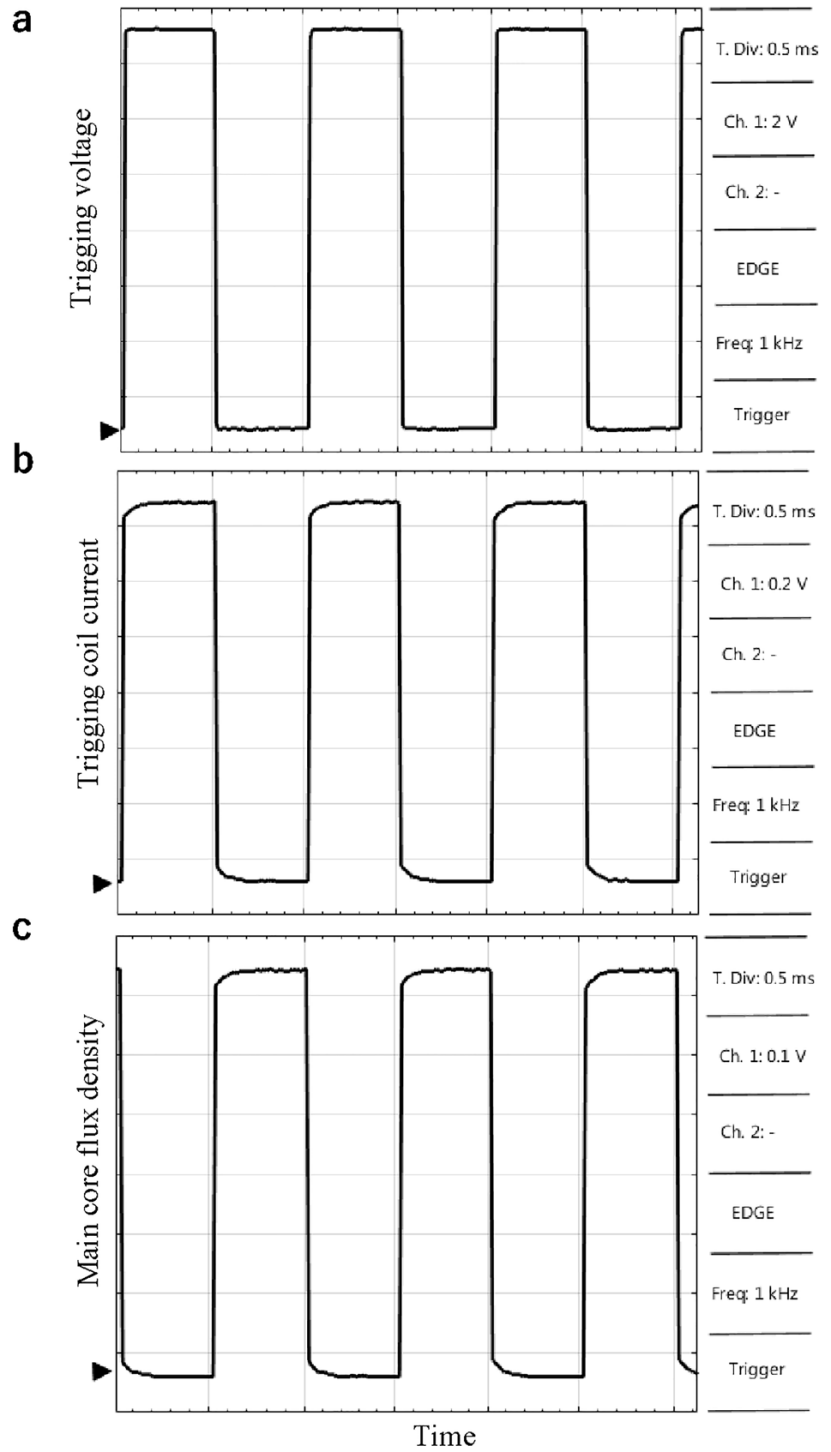
MFS DC test. (**a**) Pulse generator voltage (probe × 1). (**b**) Control coil current (probe × 1). (**c**) The main core magnetic flux density (probe × 1).

In this test scenario, the control coil is excited by a triggering pulse, which is generated by a laboratory pulse generator device. Then, the main coil is connected to the DC power supply. The outcome of this test is the measurement of the main core magnetic flux, which is the function of the trigging pulse. This test scenario will confirm the proposed MFS's capability to switch the main core's DC flux. Figure 17a illustrates the trigging voltage whose frequency is 1 kHz, the duty cycle is 50%, and the pulse magnitude is nearly 15 V. At the same time, Fig. 17b shows the current of the control coil current which its maximum magnitude reaches almost 14 µA and Fig. 17c demonstrates magnetic flux density of the main core that its magnitude takes place in nearly 0.7 T which is measured by the magnetic sensor and math function of the oscilloscope device.

This test agrees with MFS's simulation results in Fig. 12. Moreover, this test proves that when the current is excited in the control coil, MFS behaves as an open switch that declines the main core flux to zero. In contrast, when the current of the control coil is interrupted, MFS behaves as a close switch and passes the maximum flux of the main core.

### MFS AC magnetic flux switching test (second scenario)

**Figure 18:**
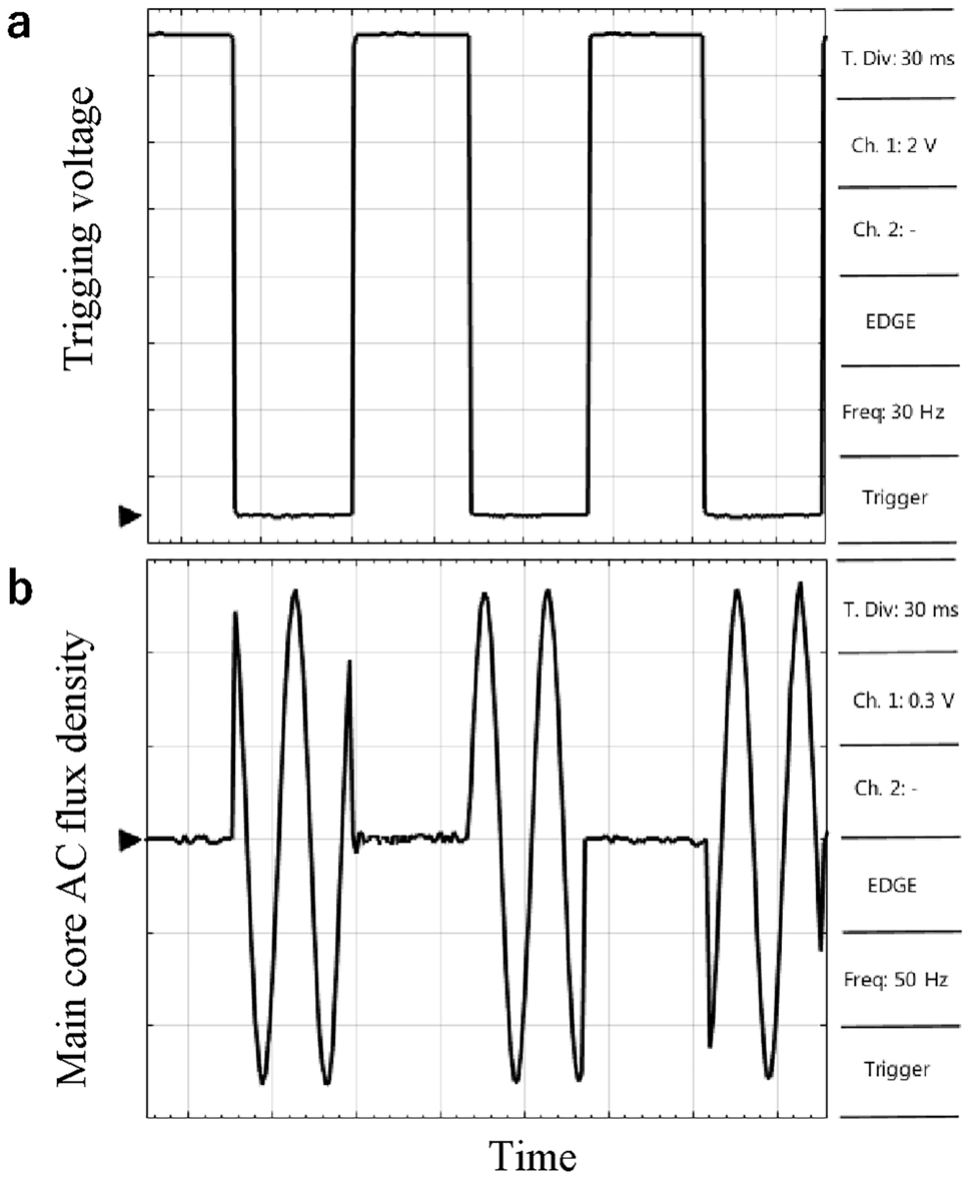
MFS AC test. (**a**) Pulse generator voltage (probe × 1). (**b**) AC flux density of the main core (probe × 1).

In the next test scenario, the main coil is connected to the AC source, and it is proposed to switch the AC flux of the main core by exciting the control core of the MFS. Therefore, the control coil is connected to the pulse generator and adjusted in a 30 Hz 50% duty cycle. As results shown in Fig. 18, when the pulse generator provides a maximum voltage for the control coil, MFS behaves as an open switch, and the AC flux of the main core reaches almost zero. However, when the trigging logic pulse is zero, the AC flux of the main core jumps to its maximum, which is 0.8 T.

These results confirm MFS's operation as a bidirectional switch and simulation results, as provided in Fig. 13.

### Prospective of using MFS in energy systems

In the presently used energy system, control of the magnetic flux in the converters, power system reactors, transformers, and electro-mechanical machines (like wind power generators, synchronous machines, and electric motors) is vital. The solution is to utilize power electronics in combination with control systems. Undoubtedly, using power electronics is very practical; however, it has both challenges and advantages. The challenges of using the power electronic switches are difficulty in designing high voltage switches (considering their reliability, difficulty of control, and solid-state power loss). Moreover, switches are always at risk of voltage stress and power system transients. These challenges introduce difficulty in using solid-state power electronic switches in some energy systems.

From a prospective point of view, MFS is a concept that can be used to construct power system components. Using MFS, it will be possible to create power components as follows: MFS power convertor (without using power electronic switches), controllable series and parallel reactors, controllable fault current limiters, controllable generators (DFIGs, PMSGs, synchronic machines), controllable motors.

## Conclusions

This paper introduces and assesses the innovative Magnetic Flux Switch (MFS) through a comprehensive investigation involving analytical studies, Finite Element Method (FEM) simulations, and experimental laboratory testing. The MFS employs a groundbreaking magnetic approach that enables the direct switching of magnetic flux within the core. This approach represents a departure from the conventional practice of utilizing power electronic switches to modulate current to control the core's magnetic flux. The achieved advantages from the MFS are listed as follows:

- the MFS magnetic flux variation ratio is more than 1000
- it needs a meager Trigging voltage and current
- Low trigging power loss around 0.1 W
- Simple control system including low current, low voltage pulse generator
- High switching speed due to control circuit low rising and falling time constant
- No mutual inductance between the main and control coil

The experimental results affirm the effectiveness of the MFS in seamlessly and efficiently toggling the magnetic flux of the main core in both directions, requiring only a minuscule amount of control current. This allows alternating the main core's magnetic flux from approximately 1 mT to 700 mT with a mere few microamps of control current. This pioneering concept heralds a novel paradigm by serving as a magnetic transistor, simplifying the design and operation of numerous components within energy systems that traditionally rely on power electronic switches.

## Methods

### Finite elements method simulation

This simulation is carried out in the ANSYS-Maxwell software platform version 2022 R1. In the first step, the geometry of MFS is designed, including cores (main and control cores), coils, and a terminal for each coil, and the H = 0 boundary considers a sphere with a 200 mm diameter. The transient analysis is run using an external circuit, including a sinusoidal and pulse source.

### Material and properties

The used material for the laboratory MFS core (for both main and control cores) is iron powder (Fe) 99%. The main core cross section is 255 mm^2^, and the control core cross section is 150 mm^2^. The cores' saturating magnetic flux density is 1.8 T, and the maximum withstand temperature under normal operation is 70 degrees Celsius. The maximum operation frequency of the core is 100 kHz. The wires used for the MFS prototype are pure copper (Cu) with a resin isolator. The wire cross-section for the main coil is roughly 0.35 mm^2^, and the control coil's is 0.28 mm^2^. The coils are built up of PVC sheets.

### MFS construction and assembly

To cut the cores in the determined geometry to verify the FEM simulation of MFS, the computer numerical control (CNC) based wire-cutting machine (high accuracy cutting machine) is used to decline the air gap of MFS junction to zero. Before assembling the cores, the main and control core wires are wounded around the formers assembled by cores. Then, the main and control cores are assembled together by mechanical force. Additionally, the main core has a separate coil to measure the signals. In the final step, all the coils are connected to the terminals.

### Laboratory setup and measurement

In the laboratory setup, the control coil is connected to a pulse generator device with a variable voltage range between 1 and 20 V and a frequency range between 10 Hz and 1 MHz. Also, variable AC and DC sources excite the main coil with sinusoidal and direct current. The voltage and current of the MFS are directly measured by voltage measurement of the oscilloscope device model OWON DS 5032E. The flux of the cores is measured by an external measurement coil applied around the cores. The voltage of the measurement coil is changed to the magnetic flux of the cores by applying the equation $\varphi (t)=\frac{1}{N}\int V(t)dt$ , where *N* and *V* are the turns and voltage of the measurement coil, respectively.

### Supplementary Information

Supplementary Information.

## Data Availability

The datasets used and/or analyzed during the current study are available from the corresponding author upon reasonable request.
