# Supplementary material for: The principle of magnetic flux switch
Source: Sci Rep. 2024 Apr 18;14:8990. doi: 10.1038/s41598-024-59721-0 (PMC11026425; doi:10.1038/s41598-024-59721-0)
Supplement: Supplementary file 1 — Supplementary Information. [file 41598_2024_59721_MOESM1_ESM.pdf]

The Features and Provided Advantages of MFS Over The State of the Art

Considered features to judge the MFS advantages

Feature 1: Ability to vary the magnetic flux of a coil wound on a core from the minimum amount to the maximum with a large magnetic flux variation ratio.
Description: The MFS should demonstrate the capability to efficiently modulate the magnetic flux within a wide range, ensuring flexibility and adaptability in various applications.

Feature 2: Triggering with low current and reasonable control voltage.
Description: Effective triggering with minimal current and voltage requirements is essential for energy efficiency and reliable operation of the MFS, reducing power consumption and enhancing control precision.

Feature 3: Low triggering power loss.
Description: Minimizing power loss during the triggering process is crucial to ensure optimal energy utilization and prevent unnecessary heat dissipation, thereby improving overall efficiency and performance.

Feature 4: Simple control circuit.
Description: A straightforward and easily implementable control circuit simplifies the design and integration of the MFS into different systems, reducing complexity, cost, and potential points of failure.

Feature 5: High-speed switching operation (in the frequency range of power applications).
Description: The MFS should exhibit rapid switching capabilities suitable for operation within the frequency range of power applications, ensuring responsiveness and reliability in dynamic environments.

Feature 6: Insulated control system (having no magnetic coupling and mutual inductance).
Description: An insulated control system devoid of magnetic coupling and mutual inductance ensures isolation and prevents unwanted interference between different components, enhancing safety and stability in operation.

Advantages of MFS over the state-of-the-art

The table below outlines the primary features of the MFS alongside four other flux control devices discussed in our manuscript. This table also visually illustrates all concepts. By considering each component's presented features, the MFS's advantages become readily apparent. The table is as follows:

<table>
<tr><th>Compared technologies</th><th>Features</th></tr>
<tr><td>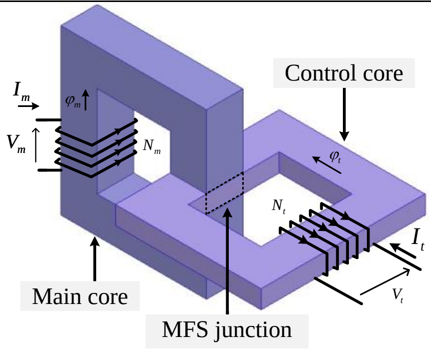

Magnetic flux switch (MFS)</td><td>

- magnetic flux variation ratio: more than 1000
- Trigging voltage: 15 V, Trigging current: 10-100 μA
- Trigging power loss: nearly 0.1 Watt
- Simple control system including low current, low voltage pulse generator
- High switching speed due to control circuit low rising and falling time constant
- No mutual inductance between the main and control coil

</td></tr>
<tr><td>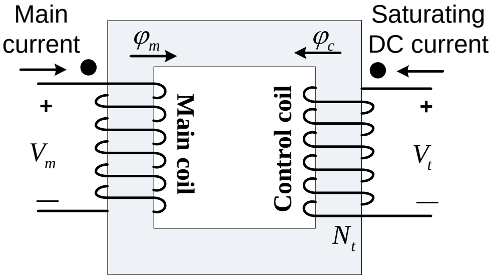

Magnetic amplifier (MA)</td><td>

- Magnetic flux variation ratio: more than 1000
- Trigging voltage: 100-1000 V Trigging current: 10-1000 A
- Trigging power loss: several kilowatt
- Complex control system including high current source and large energy observer
- Low switching speed due to the high time constant of the control circuit
- High mutual inductance between the main and control coil

</td></tr>
<tr><td>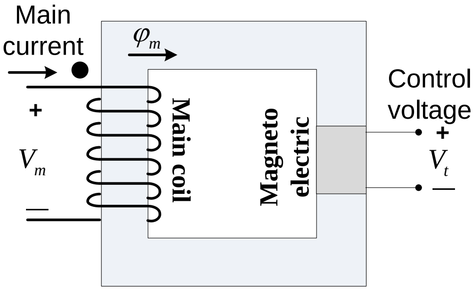

Magnetic flux valve (MFV)</td><td>

- Magnetic flux variation ratio: less than 100
- Trigging voltage 15-30, Trigging current: 1-10 A
- Trigging power loss: 10-500 Watt
- Control system complexity: very complex due to composite material design
- Switching speed: fast due to the fast operation of the piezoelectric
- No Mutual inductance between the main coil and the magnetoelectric

</td></tr>
<tr><td>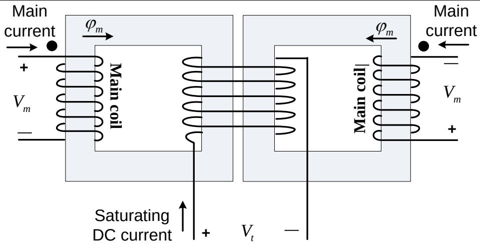

Decoupled magnetic amplifier (DMA)</td><td>

- Magnetic flux variation ratio: more than 1000
- Trigging voltage: 100-1000 V Trigging current: 10-1000 A
- Trigging power loss: several kilowatt
- Complex control system including high current source and large energy absorber
- Low switching speed due to the high time constant of the control circuit
- Low mutual inductance between the main and control coil

</td></tr>
<tr><td>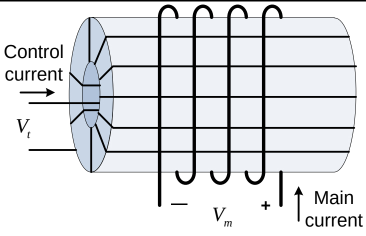

Orthogonal flux gate (OFG)</td><td>

- Magnetic flux variation ratio: less than 10
- Trigging voltage: 10-1000 V, Trigging current 10-1000 A
- Trigging power loss: more than 10 W
- Complex control system including high current source and large energy observer
- Low switching speed due to the high time constant of the control circuit
- Low mutual inductance between the main and control coil

</td></tr>
</table>

Subsequently, considering the advantages of MFS compared to other technologies, the MFS concept can be used to enhance various applications as power components that conventionally operate based on power electronic and solid-state switches. These components can be as follows: MFS power convertor (without using power electronic switches), controllable series and parallel reactors, controllable fault current limiters, controllable generators (DFIGs, PMSGs, synchronic machines), and controllable motors.
